# Controlled Synthesis of Cu and Cu_2_O NPs and Incorporation of Octahedral Cu_2_O NPs in Cellulose II Films

**DOI:** 10.3390/nano8040238

**Published:** 2018-04-14

**Authors:** Alireza Eivazihollagh, Magnus Norgren, Christina Dahlström, Håkan Edlund

**Affiliations:** FSCN, Surface and Colloid Engineering, Mid Sweden University, SE-851 70 Sundsvall, Sweden; christina.dahlstrom@miun.se

**Keywords:** copper nanoparticles, cuprous oxide nano-octahedrons, hybrid material, regenerated cellulose, chemical reduction, chelating agent, surfactant

## Abstract

In this study, Cu and Cu_2_O nanoparticles (NPs) were synthesized through chemical reduction of soluble copper-chelating ligand complexes using formaldehyde as a reducing agent. The influence of various chelating ligands, such as ethylenediaminetetraacetic acid (EDTA), diethylenetriaminepentaacetic acid (DTPA), and a surface-active derivative of DTPA (C_12_-DTPA), as well as surfactants (i.e., hexadecyltrimethylammonium bromide (CTAB), dodecyltrimethylammonium chloride (DoTAC), sodium dodecyl sulfate (SDS), and dimethyldodecylamine-N-oxide (DDAO)), on morphology and the composition of produced NPs was investigated. In the absence of surfactants, spherical copper particles with polycrystalline structure could be obtained. X-ray diffraction (XRD) analysis revealed that, in the presence of EDTA, the synthesized NPs are mainly composed of Cu with a crystallite size on the order of 35 nm, while with DTPA and C_12_-DTPA, Cu_2_O is also present in the NPs as a minority phase. The addition of ionic surfactants to the copper–EDTA complex solution before reduction resulted in smaller spherical particles, mainly composed of Cu. However, when DDAO was added, pure Cu_2_O nano-octahedrons were formed, as verified by high-resolution scanning electron microscopy (HR-SEM) and XRD. Furthermore, a hybrid material could be successfully prepared by mixing the octahedral Cu_2_O NPs with cellulose dissolved in a LiOH/urea solvent system, followed by spin-coating on silica wafers. It is expected that this simple and scalable route to prepare hybrid materials could be applied to a variety of possible applications.

## 1. Introduction

Controllable synthesis of metal and metal oxide nanoparticles (NPs) with a specific morphology and chemical composition has been at the forefront of nanoscience and nanotechnology research [1,2,3,4]. The functionality of these NPs depends on their size, shape, composition, and crystallinity, all of which require a careful synthetic strategy [2]. As one of the most inexpensive and versatile metals, Cu and Cu_2_O NPs with tailored shapes have attracted considerable attention recently. Their distinctive properties give rise to utilization in numerous potential applications, including electronic devices, optics, catalysis, photocatalysis, gas sensors, solar cells, wastewater treatment, and biomedical materials [1,2,3,4,5,6,7,8,9,10,11,12,13,14].

To synthesize Cu or Cu_2_O NPs with controlled morphology and composition, several approaches, such as microemulsion, polyol, solvothermal, sonochemical [4], electrodeposition [5], hydrothermal [6], and wet chemical reduction [1,2,3,7,8,9,10,11,12,13,14] techniques, have been employed. Among these, the chemical reduction method is a simple, low-cost, and scalable technique that only requires a reducing agent to reduce copper salt to metallic Cu or cuprous oxide Cu_2_O NPs. Reducing agents used for this purpose include sodium borohydride [1,7,8], hydrazine [3,9,10], hydroxylamine [11,12], glucose [13,14], ascorbic acid [14,15], sodium ascorbate [3], or formaldehyde [2,8]. Surfactants such as sodium dodecyl sulfate (SDS) [11,12], hexadecyltrimethylammonium bromide (CTAB) [12], and octylphenyl ether (Triton X-100) [14], and polymers such as polyvinylpyrrolidone (PVP) [1,13], polyethylene glycol (PEG) [15], and regenerated cellulose [2,10], are also used to stabilize fabricated NPs and to control their shape and size. Although great advances have been made in the preparation of Cu and Cu_2_O NPs, the synthesis strategies are time-consuming and costly due to the number of steps required, and the overall complexity of the preparation process still requires further improvements.

Herein, we present a water-based approach for the synthesis of monodisperse Cu and Cu_2_O NPs from copper sulfate solutions via reduction with formaldehyde under alkaline conditions at room temperature. The NP morphology and composition was controlled by various surfactants and chelating agents. A novel cellulose-based hybrid material was also prepared by spin-coating of octahedral Cu_2_O NPs dispersed in a water-based cellulose solution containing LiOH/urea. This low-cost and scalable approach for the controlled synthesis of Cu and Cu_2_O NPs could be extended to the development of new classes of hybrid materials with improved functionality.

## 2. Materials and Methods

### 2.1. Materials

The chelating agents, ethylenediaminetetraacetic acid (EDTA) and diethylenetriaminepentaacetic acid (DTPA), were supplied by Sigma-Aldrich (Stockholm, Sweden). The chelating surfactant 2-dodecyldiethylenetriaminepentaacetic acid (C_12_-DTPA) was delivered by Syntagon AB (Stockholm, Sweden) [16]. The chemicals hexadecyltrimethylammonium bromide (CTAB), dodecyltrimethylammonium chloride (DoTAC), sodium dodecyl sulfate (SDS), dimethyldodecylamine-N-oxide (DDAO), trimethylamine N-oxide dehydrate and tetramethylammonium hydroxide pentahydrate were of analytical grade, obtained from Sigma-Aldrich (Stockholm, Sweden), and used without further purification. CuSO_4_·5H_2_O, NaOH, LiOH·H_2_O, urea, and formaldehyde (CH_2_O, 36 wt % solution) were supplied by VWR International (Umeå, Sweden). Cellulose in the form of dissolving pulp with viscosity of 450 mL g^−1^ was supplied by Domsjö Fabriker (Örnsköldsvik, Sweden). The cellulose was ground and dissolved directly in aqueous 4.6 wt % LiOH/15 wt % urea solution precooled to –12 °C to prepare a transparent 1 wt % cellulose solution [17]. The water used for the preparation of samples was of Milli-Q grade.

### 2.2. Synthesis of Cu and Cu_2_O NPs

Cu and Cu_2_O NPs were synthesized using the chemical reduction procedure adapted from a recently reported method [2]. In a typical procedure, a 0.01 M copper complex solution CuSO_4_: chelating ligand (i.e., EDTA, DTPA, and C_12_-DTPA) was prepared. To investigate the effect of different surfactants on the synthesized NPs, a 0.01 M copper complex solution CuSO_4_: EDTA: surfactant (i.e., CTAB, DoTAC, SDS, and DDAO) was used. In addition, the influence of trimethylamine N-oxide, resembling the hydrophilic headgroup of DDAO, and tetramethylammonium hydroxide, as a non-oxygen containing nitrogen compound, on the synthesis was also studied. In all experiments, the alkaline copper complex solution (containing 0.012 mol NaOH in 50 mL) was stable without any indication of Cu(OH)_2_ precipitation. To synthesize Cu and Cu_2_O NPs, about 6.5 mL of formaldehyde (CH_2_O) and NaOH (pellets, 0.125 mol) were continuously added into 50 mL of the above complex solution. The NPs synthesis was completed in under 20 min. The product was separated by simple vacuum filtration, washed thoroughly with Milli-Q water, and air dried at room temperature before further characterization. The synthetic procedure was performed under constant magnetic stirring at ambient temperature.

### 2.3. Preparation of Cu_2_O NPs-Cellulose Hybrid Films

First, 0.007 g of synthesized octahedral Cu_2_O NPs were dispersed in 1 g of an aqueous 4.6 wt % LiOH/15 wt % urea solution. Then, 0.27 g of the resulting suspension was carefully mixed with 0.27 g of 1 wt % cellulose solution in 4.6 wt % LiOH/15 wt % urea. The resulting mixture was immediately spin-coated at 3400 rpm for 40 s onto silica wafers (ca. 10 mm × 10 mm) that had been pretreated in 1 M NaOH, washed in Milli-Q water and ethanol, and finally dried by a stream of nitrogen. The prepared Cu_2_O NPs-cellulose films were placed in ethanol for 1 h and then in Milli-Q water at room temperature for 2 h to regenerate the cellulose and remove LiOH and urea. Thereafter, the hybrid material was air dried at room temperature and collected for further analysis.

### 2.4. Characterization

The samples were coated with approximately 2 nm thick layer of iridium to obtain an electrically conductive surface. Digital images of the sample surface were acquired using a high-resolution scanning electron microscope (HR-SEM), TESCAN MAIA3 model 2016. Secondary electron images (SEI) were generated using 5 kV accelerating voltage and an in-lens detector. X–ray diffraction was carried out at room temperature using a Bruker D2 Phaser diffractometer with Cu K_α_ radiation (wavelength 1.54 Å) in θ–2θ geometry. The sample was placed on a silicon single crystal especially cut to provide a low background free from any interfering diffraction peaks.

## 3. Results and Discussion

**Figure 1:**
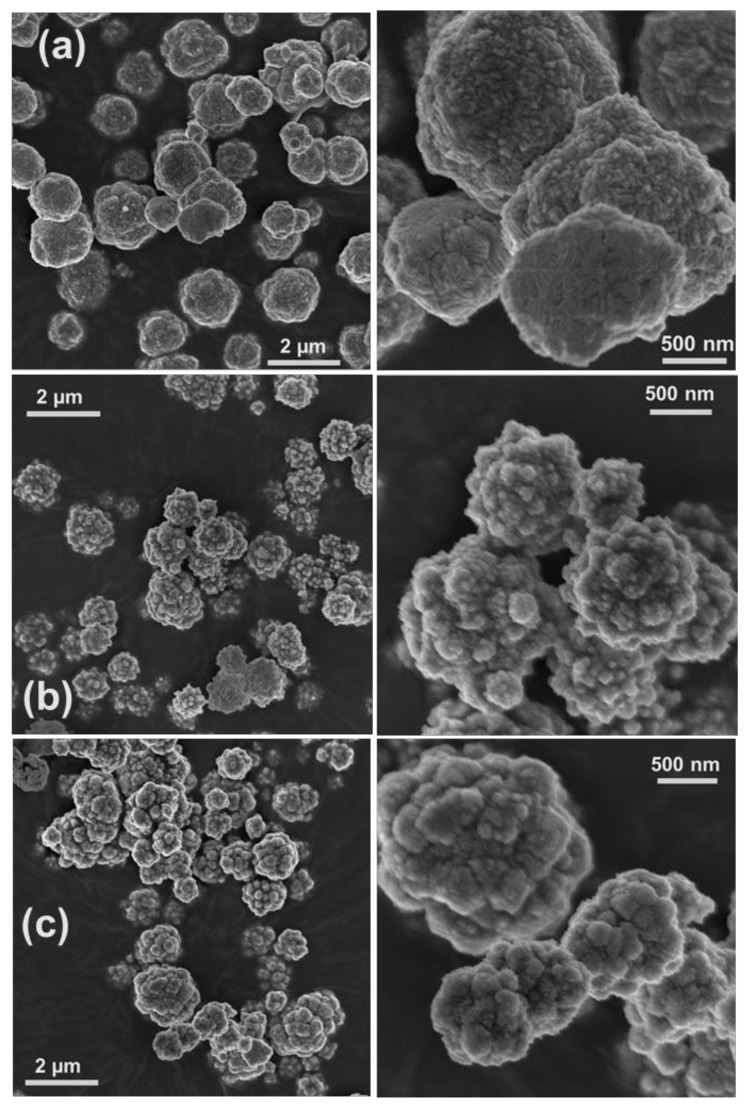
HR-SEM images of materials obtained in 0.01 M Cu(II) solutions containing (**a**) 0.01 M EDTA, (**b**) 0.01 M DTPA, and (**c**) 0.01 M C_12_-DTPA. The images show the polycrystalline structure of spherical copper particles of 1–1.5 µm, synthesized in solution (**a**) and copper particles of 800 nm–1.2 µm, formed in solutions (**b**) and (**c**). The images on the right-hand side are of synthesized materials (**a**–**c**) at higher magnifications.

**Figure 2:**
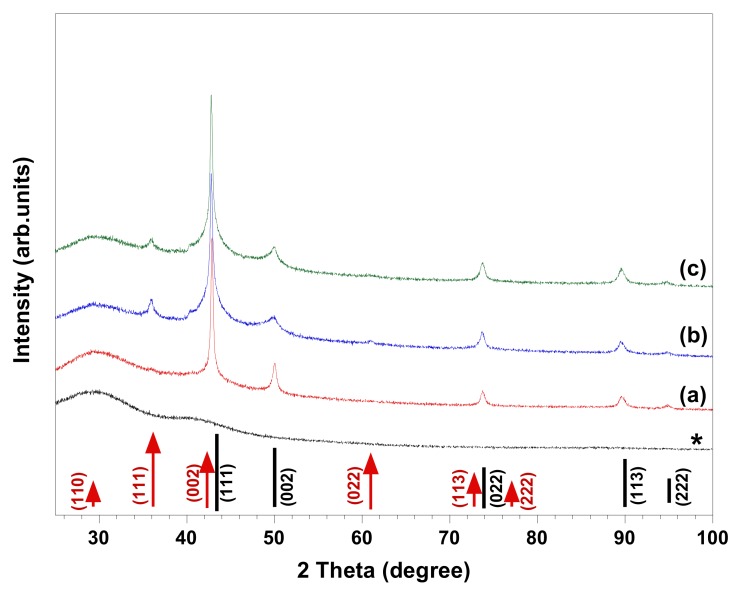
XRD patterns of materials obtained in 0.01 M Cu(II) solutions containing (**a**) 0.01 M EDTA, (**b**) 0.01 M DTPA, and (**c**) 0.01 M C_12_-DTPA. The positions of the expected Bragg peaks from copper (black lines), and cuprous oxide (red arrows) are marked and labeled with their respective Miller indices. The diffraction pattern denoted by * in the plot stems from the type RA Millipore membrane filter used to separate synthesized material by simple vacuum filtration.

We have recently reported a route for in situ synthesis of spherical Cu/Cu_2_O NPs in a cellulose matrix through reducing chelated 0.01 M copper ions by CH_2_O under ambient conditions [2]. We considered this synthetic method a good starting point to evaluate the influence of various chelating ligands and surfactants on the synthesized NPs’ morphology and chemical composition. Numerous chemical syntheses of Cu and Cu_2_O NPs have made use of a variety of chelating agents such as EDTA [2,8], nitrilotriacetic acid (NTA) [2], tartrate [8], citrate [8,13], and NH_3_ [9] to suppress the formation of Cu(OH)_2_ at high pH, which is required for CH_2_O to act as a reducing agent [2,8]. It has been claimed that different chelating agents and the changes in their concentration with respect to copper and base concentrations, as well as changing the reducing agent, could influence the morphology and phase composition of synthesized material [8]. HR-SEM images and XRD patterns of the materials synthesized in copper complex solutions comprising different chelating ligands (i.e., EDTA, DTPA, and C_12_-DTPA) are given in Figure 1 and Figure 2, respectively. The images show the polycrystalline structure of spherical Cu particles (Figure 1). Ostwald growth and ripening may be responsible for the formation of Cu spheres with polycrystalline structure [3,8]. In addition, this could be related to the fast nucleation of Cu due to the strong reducing ability of the reductant used [3]. In Figure 2, XRD analyses indicated that in presence of EDTA, the synthesized material is composed of Cu and possibly a trace amount of Cu_2_O, while in the case of DTPA and its surface-active derivative C_12_-DTPA, Cu_2_O is also present as a minority phase. As seen in the HR-SEM images, the copper particles obtained in solutions containing DTPA and C_12_-DTPA have rougher surfaces compared to the ones synthesized in the presence of EDTA (Figure 1). This rough surface of polycrystalline spherical particles may be attributed to incomplete Ostwald ripening. However, this could also be related to the presence of Cu_2_O in samples obtained in solutions containing DTPA and C_12_-DTPA. Since the composition and morphology of copper particles synthesized in solutions containing DTPA and C_12_-DTPA are similar to each other, it can be concluded that the surface properties of chelating surfactant C_12_-DTPA had no noticeable influence on the resulting material under the experimental conditions studied. To our knowledge, there have been no studies regarding the use of DTPA and its surface-active derivative C_12_-DTPA for the synthesis of copper particles via the chemical reduction method. As the prepared samples are mainly composed of Cu, the crystallite size of copper in the samples was estimated from the line broadening of the Cu (111) diffraction peak (Figure 2) that had the highest intensity, using the Scherrer equation under the assumption that the contributions of chemical disorder and mechanical strain to the line broadening of peaks are negligible. The Cu crystallite size of the polycrystalline spherical particles obtained in complex solutions containing EDTA, DTPA, and C_12_-DTPA was calculated to be 37, 30, and 30 nm, respectively. In addition, the HR-SEM images in Figure 1 show that the copper spheres synthesized in solutions containing DTPA and C_12_-DTPA are slightly smaller than the ones obtained in the presence of EDTA. The decrease in size can be explained by the larger negative shift of the copper reduction potential [8] in solutions containing DTPA and C_12_-DTPA compared to the solution containing EDTA, as a result of the higher stability constant (log_10_ *K*) of CuDTPA^3−^ complexes compared to CuEDTA^2−^ and Cu(OH)EDTA^3−^ complexes, which are the predominant copper species in their alkaline solution [18].

**Figure 3:**
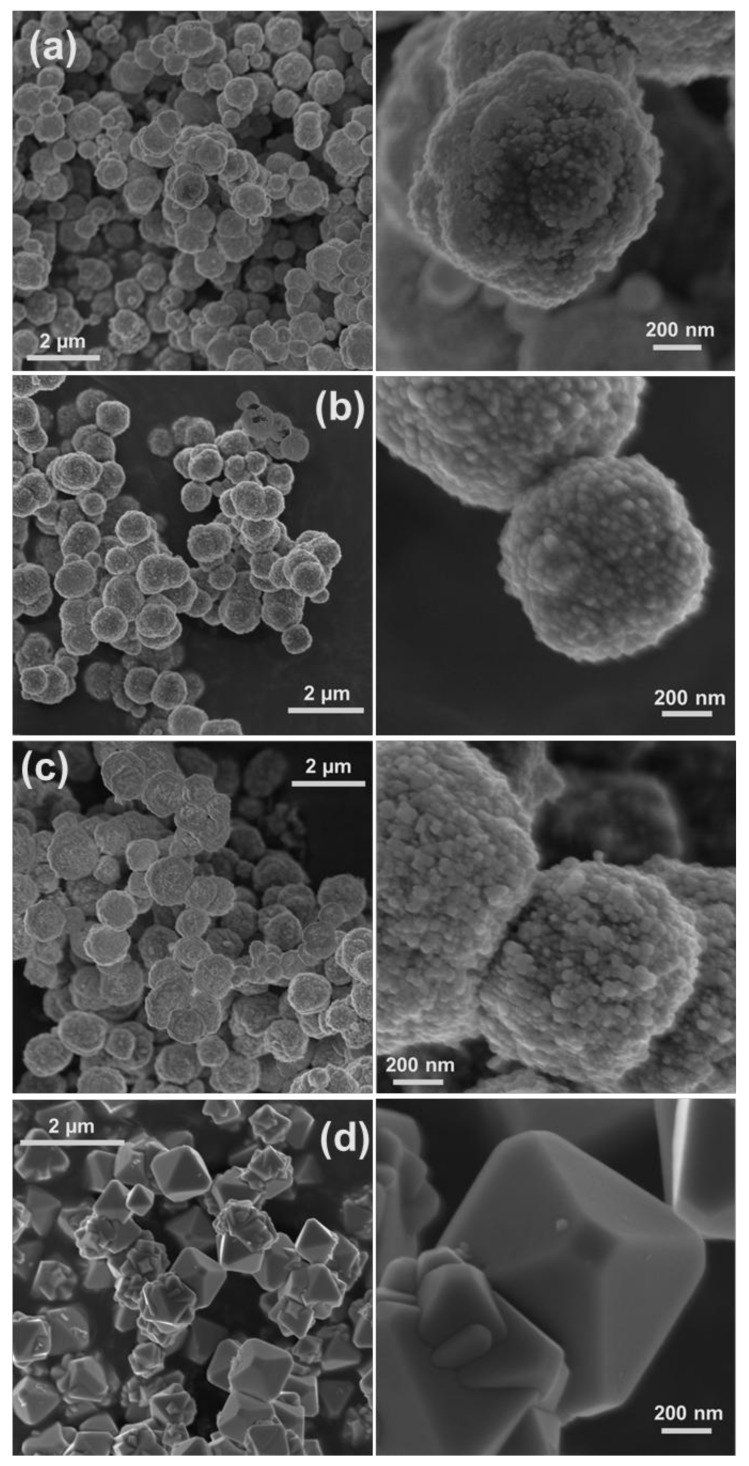
HR-SEM images of synthesized materials in 0.01 M Cu(II)—0.01 M EDTA solutions containing (**a**) 0.01 M CTAB; (**b**) 0.01 M DoTAC; (**c**) 0.01 M SDS; and (**d**) 0.01 M DDAO. The images show the polycrystalline structure of spherical copper NPs of 600–900 nm, synthesized in solutions (**a**–**c**) and octahedral cuprous oxide NPs of 500–900 nm, formed in solution (**d**). The images on the right-hand side are of synthesized materials (**a–d**) at higher magnifications.

**Figure 4:**
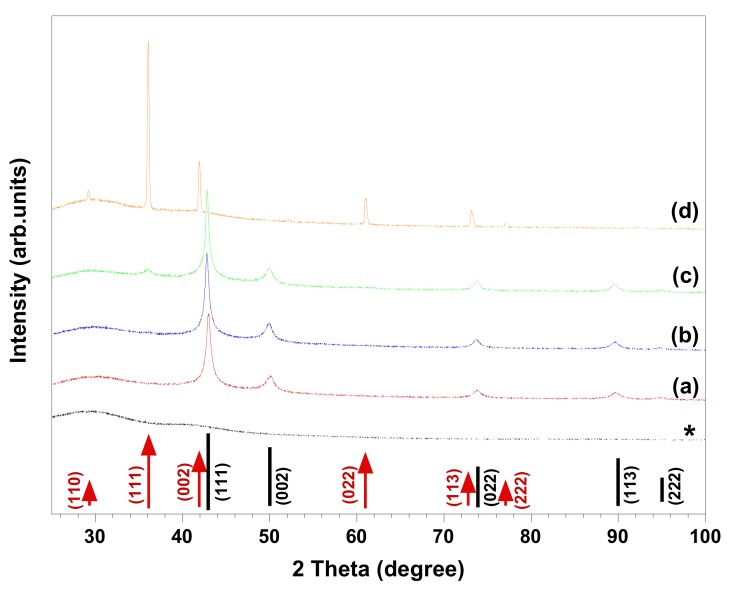
XRD patterns of synthesized materials in 0.01 M Cu(II)—0.01 M EDTA solutions containing (**a**) 0.01 M CTAB, (**b**) 0.01 M DoTAC, (**c**) 0.01 M SDS, and (**d**) 0.01 M DDAO. The positions of the expected Bragg peaks from copper (black lines), and cuprous oxide (red arrows) are marked and labeled with their respective Miller indices. The diffraction pattern denoted by * in the plot stems from the type RA Millipore membrane filter used to separate synthesized material by simple vacuum filtration.

In the chemical reduction-based route, surfactants and polymers have been effectively used as shape- and/or size-controlling agents to produce NPs with specific morphology and as stabilizers to prevent the aggregation of particles [1,2,10,11,12,13,14,15]. We have previously observed that regenerated cellulose (cellulose II) serves both as stabilizer and template, resulting in the synthesis of copper NPs in the size range of 200–500 nm [2], which were smaller than the synthesized copper particles in the present study (Figure 1). To investigate the influence of surfactants on the morphology and composition of synthesized materials, control experiments were performed in which the same chemical amount of cationic CTAB and DoTAC, anionic SDS, and zwitterionic DDAO were added to a 0.01 M CuSO_4_: EDTA solution. Figure 3 and Figure 4 show HR-SEM images and XRD patterns of the materials produced in the abovementioned solutions. The HR-SEM images in Figure 3a–c clearly reveal that the studied ionic surfactants affected the synthesis of NPs almost to the same extent, resulting in polycrystalline spherical NPs of 600–900 nm, which were relatively smaller than the particles synthesized in absence of surfactant (Figure 1a). It should be noted that both cationic surfactants have similar headgroups, while CTAB (C_16_-) has a longer hydrocarbon chain length than DoTAC (C_12_-). In addition, the counterions of CTAB and DoTAC are Br^−^ and Cl^−^, respectively, which also may affect the morphology of obtained NPs [7]. Through the comparison of the representative XRD patterns in Figure 4a,b and the pattern in Figure 2a, it is concluded that the cationic surfactants did not alter the phase composition, which is found to consist of metallic copper. However, in the presence of anionic SDS, the XRD pattern indicated that Cu_2_O was present as a minority phase in the obtained material (Figure 4c). On the other hand, when zwitterionic DDAO was adopted, pure Cu_2_O nano-octahedrons were formed, as verified with HR-SEM and XRD (Figure 3d and Figure 4d).

Several synthetic protocols for the fabrication of faceted Cu_2_O NPs via chemical reduction have been reported. Within these approaches, the synthesis of pure faceted Cu_2_O NPs was claimed to be achieved by controlling the atmosphere (e.g., O_2_) and temperature of reaction media, the concentrations of Cu(II), OH^−^, chelating ligand, and reducing agent, as well as the ratios between them in solution, and/or by changing the reducing agent and chelating ligand [1,3,7,8]. In some procedures, surfactants and polymers were also employed as shape-controlling agents and stabilizers [1,7]. However, to our knowledge no report describing the application of surfactants to control the phase composition of synthesized copper NPs has been presented. In the present study, we observed that by using DDAO, the synthesis of pure faceted Cu_2_O NPs (i.e., octahedron shapes) could be achieved (Figure 3d and Figure 4d). However, the synthesis in absence of the surfactant led to formation of polycrystalline spherical particles composed of Cu and possibly trace amount of Cu_2_O (Figure 1a and Figure 2a). In our study, the morphology of synthesized NPs can be expected from previous studies demonstrating that surfactant (i.e., nonionic Triton X-100) [14] and nitrogenated molecules (i.e., EDTA, and NH_3_) [8,9] could stabilize Cu_2_O {111} planes during crystal growth, resulting in the formation of octahedral Cu_2_O NPs. DDAO has been previously employed to produce supported copper oxide (i.e., Cu_2_O, and CuO) on magnesium oxide using a surfactant-mediated hydrothermal approach [6]. In addition, trimethylamine N-oxide was used as an oxygen-transfer reagent for controlled oxidation of amorphous core-shell Fe–Fe_3_O_4_ NPs to obtain hollow Fe_3_O_4_ NPs using a solution-phase method [19]. To the best of our knowledge, there have been no studies regarding the use of DDAO to promote the synthesis of pure faceted Cu_2_O NPs (i.e., octahedron) via chemical reduction. To investigate the role of DDAO in the formation of faceted Cu_2_O NPs, synthesis was also performed in which the same chemical amount of trimethylamine N-oxide, resembling the hydrophilic headgroup of DDAO, and tetramethylammonium hydroxide, as a non-oxygen-containing nitrogen compound, were added to a 0.01 M CuSO_4_: EDTA solution before reduction. The HR-SEM and XRD characterization of the obtained materials showed that polycrystalline spherical particles of Cu were produced (the results were similar to the results presented in Figure 1a and Figure 2a). It seems that both trimethylamine N-oxide and tetramethylammonium hydroxide had no influence on either the morphology or chemical composition of the synthesized particles. This suggests that the effect of DDAO on the successful synthesis of faceted Cu_2_O NPs is a consequence of the combined surface activity properties and specific headgroup functionality. In addition, the concentration dependency of DDAO additions was tested from 0.001 to 0.1 M. All DDAO concentrations studied here resulted in the formation of octahedral Cu_2_O NPs without any indication of polycrystalline Cu NPs. Since the composition and morphology of synthesized particles were unaffected by the changes in DDAO concentration, it can be concluded that this compound, which consists of amine oxide, was not the source of oxygen for copper oxide formation, but still a reaction mediator. A similar conclusion can be made when the use of trimethylamine N-oxide did not lead to copper oxide formation under comparable experimental conditions. It has been previously reported that the atmosphere under the synthesis, especially the presence of oxygen gas, could play a key role in controlling the morphology of nanocrystals [3]. In their surfactant-free approach, Xu et al. found that the morphology of Cu_2_O NPs can be tuned through reducing Cu(OH)_2_, using a suitable reducing agent (i.e., hydrazine hydrate and sodium ascorbate) under controlled atmosphere conditions (i.e., Ar and air) at ambient temperature. Cu_2_O octahedrons could be produced when the hydrazine hydrate was adopted as the reducing agent in air atmosphere [3]. In addition, the synthesis of Cu_2_O NPs with different morphology has been achieved upon slow oxidation of Cu colloidal solutions exposed to air at ambient temperature [7]. On the basis of these explanations, it would not be unreasonable to assume that a similar situation occurs in our case. Since all the experiments were performed in air atmosphere, DDAO could facilitate the contact between Cu nuclei and dissolved oxygen gas due to its surface-active properties and promote the reaction, leading to the formation of Cu_2_O NPs. However, it would be interesting to study the synthesis under controlled atmosphere conditions (i.e., Ar). It should be emphasized that the mechanism for the synthesis of octahedral Cu_2_O NPs in the presence of DDAO using the chemical reduction approach is still under investigation, and more systematic work needs to be performed to get a better knowledge of the detailed formation mechanism.

**Figure 5:**
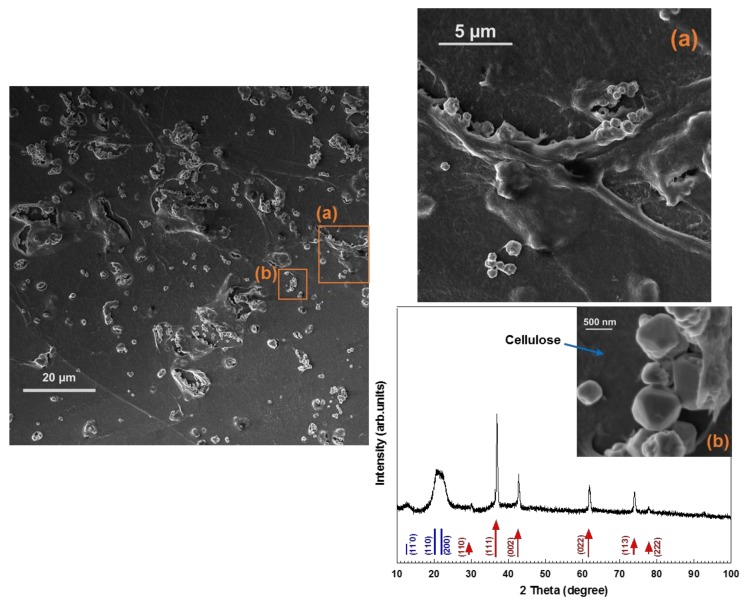
HR-SEM images and XRD pattern of hybrid material produced by spin-coating technique with cellulose solution containing octahedral Cu_2_O NPs (see Figure 3d and Figure 4d). The positions of the expected Bragg peaks from cellulose II (blue lines), and cuprous oxide (red arrows) are marked and labeled with their respective Miller indices. The images (a) and (b) on the right-hand side are high magnification HR-SEM images correspond to the rectangles (a) and (b) in the left-hand side image.

Fabrication of cellulose-based hybrid materials comprising metal and metal oxide NPs has attracted a great deal of attention in terms of practical applications and development of biocompatible hybrid materials [2,10,20,21]. The NPs’ size, morphology, composition, crystallinity, and distribution in the matrix influence the properties of hybrid material. Cellulose, as the most abundant biopolymer, has good mechanical properties and chemical resistivity in a wide range of solvents, which makes it an excellent candidate for the production of low-cost, environmentally friendly, and functional hybrid materials [2]. We recently reported on the synthesis of spherical copper NPs templated by a regenerated cellulose II matrix under alkaline aqueous reaction conditions [2]. In the present study, cellulose-octahedral Cu_2_O NPs hybrid films were fabricated by spin-coating of Cu_2_O NPs dispersed in a cellulose solution (Figure 3d and Figure 4d). In Figure 5, the XRD pattern and HR-SEM images of the hybrid material are shown. The images indicate that the Cu_2_O nano-octahedrons are well distributed throughout the regenerated cellulose matrix. As seen in Figure 5, the XRD pattern of the film shows that the hybrid material is composed of cellulose II and pure Cu_2_O. This type of hybrid material is more easily handled than non-scaffolded NPs and highly interesting, since faceted Cu_2_O NPs (i.e., octahedrons) have potential application in many fields, such as photocatalysis, solar cells, catalysis, gas sensors, and hydrogen production [1,4,8,10]. To the best of our knowledge, the only reported attempt to produce regenerated cellulose-Cu_2_O NPs hybrid material was by Tu et al. [10]. In their study, a 4 wt % cellulose solution (i.e., aqueous NaOH/urea solvent) was casted and regenerated to obtain regenerated cellulose film. To fabricate the hybrid material, the cellulose film was first placed in CuSO_4_ solution for 12 h and then transferred in NaOH solution, and finally it was immersed in hydrazine hydrate solution [10]. Our study presents a simple route to synthesize octahedral Cu_2_O NPs under 20 min for the first time, by using the zwitterionic surfactant DDAO as mediator. The NPs could thereafter be dispersed in a water-based cellulose solution that was finally spin-cast and regenerated, producing cellulose-octahedral Cu_2_O NPs hybrid films.

## 4. Conclusions

A straightforward and effective approach for the controlled synthesis of Cu and/or Cu_2_O NPs using a fast (<20 min) and simple chemical reduction technique is presented. The morphology and chemical composition of synthesized NPs could be controlled by adding different kinds of chelating agents and surfactants. We found that the spherical particles obtained in copper solution containing chelating ligands DTPA and C_12_-DTPA, as well as the particles produced in the solution containing EDTA and cationic or anionic surfactants (i.e., CTAB, DoTAC, and SDS), were smaller than the ones synthesized in a solution of copper and EDTA, and were mainly composed of Cu. For instance, the addition of ionic surfactants resulted in polycrystalline spherical NPs of 600–900 nm. In addition, DDAO, a zwitterionic surfactant, was found to mediate the formation of pure octahedral Cu_2_O NPs most likely due to enhanced oxygen gas transfer because of its surface activity and specific headgroup. A hybrid material composed of regenerated cellulose and synthesized Cu_2_O nano-octahedrons was fabricated by spin-coating. The morphology and composition of synthesized NPs can be simply controlled via this route, and our future work will focus on the synthesis of other metal/metal oxide NPs and selective functionalization of biopolymers such as cellulose using the synthesized NPs.
